# Personal identification and missing persons initiatives in Santa Catarina state, Brazil: forensic perspectives from 2019 to 2021

**DOI:** 10.1080/20961790.2022.2060653

**Published:** 2023-02-12

**Authors:** Paulo Miamoto, Clineu Julien Seki Uehara

**Affiliations:** a Forensic Anthropology Division of the Medicolegal Direction (DATF-DML), Scientific Police of Santa Catarina (PCI), Florianópolis, Brazil; b Biochemistry Division of the Forensic Laboratory Analysis Direction (DBQA-DALF), Scientific Police of Santa Catarina (PCI), Florianópolis, Brazil

**Keywords:** Forensic sciences, forensic anthropology, forensic odontology, missing persons, Brazil

## Abstract

Santa Catarina is a small, developed, and relatively safe state in South Brazil. Despite having positive social economic indicators, it still faces multiple challenges regarding forensic practices for personal identification. The objective of this paper is to discuss the recent advances and current challenges in the region, from the perspectives of anthropological and dental postmortem human identification, missing persons, and disaster victim identification (DVI) from 2019 to 2021. The recent creation of a Forensic Anthropology Sector (SAF) in the state’s official forensic institution (*Polícia Científica*—PCI) has significantly improved identification of unidentified remains and optimised resources available for DNA analysis. However, SAF is still quite understaffed, which negatively affects the recovery of skeletal material, its preparation, and the time needed for filing reports. Santa Catarina has passed legislation for missing persons in 2015, 4 years prior to the sanction of federal laws implementing the national policy for the disappeared. Nonetheless, a lack of integration between stakeholders remains a problem that PCI has tried to circumvent with the Conecta Programme, a multidisciplinary and integrated initiative between families of the missing persons, police agencies, and the Public Ministry. The programme aims to collect not only reference DNA samples, but also relevant anthropological and dental data. It also offers facial progression services in cases of disappearances that occurred many years ago. Despite a history of disasters in the state, PCI still needs to implement international DVI standards at an institutional level. Recent training on Phase 1 DVI procedures, integrated with other responding institutions, indicates better preparation for future disasters. There are many challenges ahead for Santa Catarina’s forensic institution and professionals that have yet to be addressed, but the overall situation on routine personal identification, missing persons initiatives, and DVI has improved over the last 2 years.

## Key points

Over the last 2 years, the Santa Catarina state in South Brazil has experienced significant improvements in personal identification procedures, missing persons policies, and DVI capacitation.

- The creation of the Forensic Anthropology Sector (SAF) in October 2020 (which also houses dental postmortem examinations) in the state’s official forensic institution (PCI) has established an alternative for quick, low-cost personal identification in cases of unidentified remains, in addition to optimising resources available to DNA analysis.
- PCI’s *Programme Conecta* has been locally promoting improved integration of missing persons’ stakeholders with the newly instituted national policy for the disappeared in Brazil.
- Despite having a history of disasters and a recent first integrated training on Phase 1 DVI procedures, Santa Catarina state still needs to implement DVI procedures at an institutional level.

## Introduction

Santa Catarina is the smallest of the three states of South Brazil, with a territory of 95 346 km^2^ [1] that spreads from the Atlantic Coast to the east until the border with Argentina to the west. Its population of about 7.5 million people mostly inhabits small- or medium-sized cities (86%) [2], with populations varying between 361 000 and 597 000 people in the three largest towns [3]. The indigenous population significantly decreased following the Portuguese colonisation in the 16th century, and an extensive European immigration in the 19th century significantly altered the state’s population composition: 88.1% of the population self-declared as white, 9% as mixed, 2.9% as black, and 0.2% as Asian or indigenous [4] in 2010. The climate is subtropical, with a limited but nearly annual occurrence of snow during the winter in the mountainous areas [5]. Santa Catarina is considered to be a safe and developed state of Brazil, with a very high human development index (HDI-M 0.808 in 2017) [6] and the second lowest homicide rate per 100 000 inhabitants (11.9 in 2018) in the country [7].

Regarding forensic issues, although *ad hoc* experts can be appointed as non-official experts by the judicial or police authorities to participate in the criminal investigation (which is a quite rare practice), the Brazilian Code of Criminal Procedures [8], the Brazilian Criminal Code [9], and specific legislation [10] establish that the analysis of human remains be conducted by official experts working at the Medicolegal Institute (IML) after passing a public admission examination. In Santa Catarina, the Medicolegal Direction (DML, formerly known as IML) is one of four Directions that form a larger organisation known as Scientific Police (*Polícia Científica*—PCI, formerly known as *Instituto Geral de Perícias—*IGP). This is the state’s permanent institution for official forensic expertise of non-federal cases, and is responsible for various types of criminal forensic analysis, civil and criminal identification, and research and development of forensic knowledge [11].

Formerly part of the Civil Police (1969–2004), the PCI is currently a 17-year-old functionally autonomous, financially independent, non-law enforcement institution that is placed directly under the Superior Collegiate of Public Security and Official Expertise Chief’s authority [12]. *A priori*, all cases of unidentified human remains are referred to one of the 27 units of the DML across the state. Cases of unidentified victims unable to be identified using friction ridge analysis are referred to the main unit in the capital, Florianópolis, where the sectors of Forensic Anthropology and Forensic Genetics are located. The sector of Forensic Genetics is housed in the Forensic Laboratory Analysis Direction (DALF), responsible for these examinations in the institution.

With above average social indicators and an autonomous official forensic institution, Santa Catarina is still part of a broader national context in which Brazil faces escalating violence with large numbers of fatalities and missing persons [13]. This article aims to discuss the recent advances and current challenges in Santa Catarina state from the perspectives of anthropological and dental postmortem personal identification, missing persons, and disaster victim identification (DVI), complementing the international discussion on the reality of the dead in Brazil.

## The Brazilian “paradox”: anthropological analysis is conducted by forensic pathologists and forensic odontologists

Brazilian legislation severely restricts the professionals performing postmortem human identification, as only three positions are admitted as official experts: forensic analysts, forensic pathologists, and forensic odontologists [10]. As pointed out by Calmon [14], experts in the IML (pathologists and odontologists) have background education and training in health sciences and their forensic interface, medicine, and odontology. Forensic analysts may have a much broader array of educational backgrounds, yet they are admitted into Institutes of Criminalistics (IC) and not the IML, examining evidence other than human remains and/or living persons. Although there are some situations where forensic analysts are transferred to an IML, there are still almost no professionals with formal education in forensic anthropology. Specific qualification in the field is not a requirement for practitioners and specific courses are still scarce. Most of the professionals, including those in Santa Catarina, work simultaneously on medicolegal/forensic dental routines besides forensic anthropological analysis. As a result, the development of the field in Brazil is completely different from the other countries of Latin America, where it has matured from the 1960s to the 1980s, in contexts of forced disappearances during previous military governments in this part of the continent. In these areas, professionals with backgrounds in forensic anthropology, bioanthropology, biology, and archaeology conduct most of the casework [14]. As Cunha [15] noted, Brazil is an exception where most of forensic anthropologists are educated as odontologists.

Although this situation may seem undesirable, Calmon [14] highlighted some recent initiatives that are effectively promoting the development of the field in Brazil, including the Laboratory of Forensic Anthropology of the Legal Medicine Centre (Centro de Medicina Legal—CEMEL) at the College of Medicine in Ribeirão Preto at the University of São Paulo (FMRP-USP), the Centre for Forensic Archaeology and Anthropology (Centro de Arqueologia e Antropologia Forense—CAAF) at UNIFESP, and Brazilian Association of Forensic Anthropology (ABRAF). In addition, studies to validate methods for estimating age, sex, and ancestry in the Brazilian population have been performed [14]. Efforts to standardise and implement good practices for anthropological analysis are underway among nationwide practitioners. For instance, ABRAF’s political action is noteworthy, as one of its vice presidents is now part of the Missing Persons’ working group in the National Secretariat of Public Safety (SENASP), linked to the Ministry of Justice and Public Safety (MJSP) [16]. The perspectives of the Brazilian context begin to be discussed with stakeholders at an international level from a local standpoint [17]. Furthermore, a recent increase of identified skeletal collections offers positive perspectives to validate and improve methods for biological profiling at a populational level [18, 19].

However, until 2019 in Santa Catarina, nearly all cases of unidentified human remains relied on either fingerprint or DNA analysis for positive identification in the DML. Anthropological and dental analyses were exceptions, with only a few dental identification cases conducted by a single professional and sparse initiatives for the development of forensic anthropology in the capital, all hindered by a heavy workforce deficit. There was no hiring of official experts since 2008 and only 35.89% of the positions were filled [20]. From this workforce, an important portion was dedicated to administrative positions, not forensic examinations. Another group was away from main activity because of medical leave. Such a situation diminished opportunities for the families of decedents to rapidly receive their remains for funeral procedures, while creating an irrational and artificial demand for expensive and time-consuming DNA analysis. In 2019, the largest hiring of forensic experts in the history of PCI (65 forensic analysts and 28 forensic pathologists, but only two fingerprint technicians and a single forensic odontologist) allowed the institution to improve personal identification practices [20], as discussed below.

## The structuring of the Forensic Anthropology Sector in 2020

The new forensic odontologist had specialised training besides an anthropological background from postgraduate studies and a humanitarian forensic mission. After he joined the institution, the dental identification routine was expanded and he voluntarily travelled across the state to perform examinations. His first case involved the quick identification of five carbonised victims of an automobile accident in the mountainous region of the state [21]. Initially, considering the *status quo* at that time, the possibility of dental examinations was disregarded. The families of the victims were informed by the local DML unit that DNA analysis would be necessary to positively identify the remains, a process that could take up to 6 or 7 months [22]. This caused outrage, as it meant that the bodies of the young victims would need to remain in the DML throughout delicate dates, such as Christmas, New Year, and Mother’s Day. However, the rapid dental identification brought relief to the families, allowed them to mourn their loved ones, and established a positive precedent within the institution. This enabled dental identifications to be performed in many other DML units.

Later, other positive identifications were conducted using not only dental treatment data, but also valid anthropological features such as anatomical osteologic variants, frontal sinuses, pathological changes, medical devices, and dental morphology [23]. Another positive precedent was set in a case of unknown skeletal remains found in 2018. No anthropological examination was carried out, but a DNA sample was collected that ultimately matched a reference in the state’s DNA database. The reference sample belonged to a mother who was searching for her two sons who disappeared, one of them aged 21 and another aged 31 in 2018. Despite the kinship between decedent and the woman, DNA analysis was not able to individualise the victim. In 2019, anthropological analysis was performed, and the skeletal remains were consistent with a male aged 18–23. This ruled out the older brother as the decedent and enabled the identification of the younger brother [24].

In cases of remains without a supposed identity, forensic anthropological analysis began to improve forensic genetics procedures by allowing new DNA profiles in Santa Catarina’s DNA database to be complemented with information about the biological profile and other distinctive features. In short, forensic anthropology and forensic odontology helped avoid conducting unnecessary DNA analysis and allowed it to provide more relevant information in the database. These advances, discussed below, and voluntary work for the revitalisation of the Laboratory of Forensic Anthropology and Odontology were recognised by the PCI administration. The Forensic Anthropology Sector (SAF) was formally instituted in October 2020 [25].

Aware of the need to follow internationally accepted standards, SAF based its procedures on the guidelines of the Latin American Association of Forensic Anthropology [26], acting on four phases: (1) the preliminary forensic investigation, involving the collection of information on the circumstances of a case as well as biological and social information of a supposed victim; (2) body and evidence recovery, with the application of archaeological techniques to search, document, and recover such materials (not to be confused with crime scene investigation out of personal identification contexts); (3) anthropological analysis, aimed at supporting personal identification; and (4) human identification itself, including both forensic anthropology and odontology techniques. The sector is based in the capital but works in cooperation with all DML units across the state. Standard Operating Procedures (SOPs) based on complementary guidelines [27–29] were also published and provided instructions for medicolegal teams outside of the capital to collect data useful for all four phases of SAF’s actions [30, 31].

### Overview of dental and anthropological postmortem examinations from 2019 to 2021

**Figure 1.**
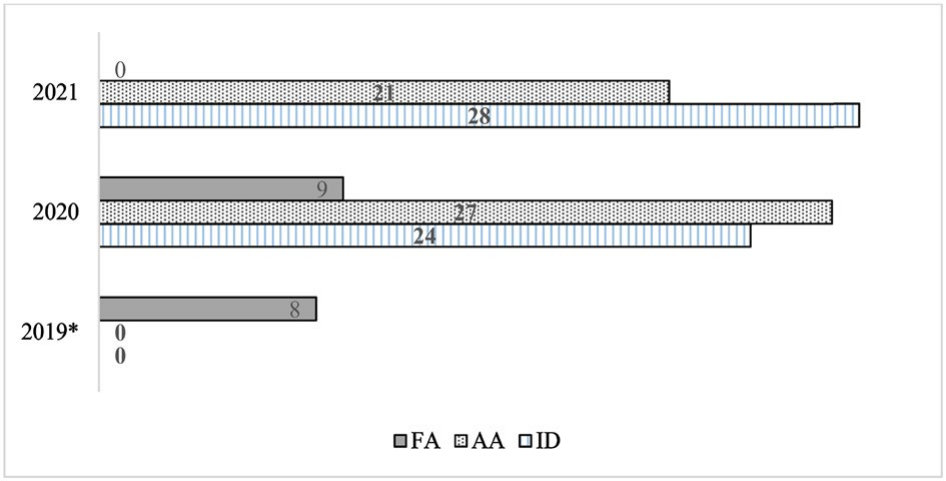
Forensic examinations for Anthropological Analysis (AA), Dental Personal Identification (ID), and Forensic Anthropology (FA, a generic denomination that did not differentiate AA and ID) from 2019 to 2021. *In 2019, five of the eight examinations were the ID of the young, carbonised victims of the car crash.

Until 2019, neither anthropological analysis (AA) nor dental personal identifications (ID) were individually accounted for in PCI’s operational system. Both examinations were generically grouped under a “Forensic Anthropology” (FA) denomination. From March 2020, these examinations started to be differentiated and composed with clearer statistics (Figure 1). Even after disregarding nine FA examinations from January to March 2020, both AA and ID examinations had an extensive annual increase (900% and 480%, respectively) relative to all FA examinations in 2019 (considering at least five of them were the ID examinations of the car crash). There was a similar demand for these examinations in 2021.

Currently, SAF examines victims in various preservation conditions (skeletonised, decayed, carbonised, mutilated, fragmented) after the examination by the forensic pathologist has been completed. A donation of equipment for maceration by Dr. Frank Marotta, forensic anthropologist from Minas Gerais state, allowed for proper examinations of skeletal material. Considering the Brazilian context, both forensic anthropology and forensic odontology postmortem examinations are housed in the same sector. Reports are signed by two professionals (odontologist and pathologist), and a multidisciplinary approach in AA examinations allows for discussion and eventually establishment of cause (but not manner) of death if the pathologist’s examination is inconclusive.

In addition to including the DNA profiles of new unknown remains in the state’s DNA database (which is integrated with the Brazilian National DNA Database) [32], one of the most significant improvements implemented by AA examinations is the analysis of trauma and its relevance for stakeholders investigating, prosecuting, defending, or judging a case. Maceration and proper analytical methodology [33–35] enabled traumatic dynamics to be evidenced and conclusively reported.

For ID examinations, reconciliation of dental data is complemented by anthropological identifiers, such as medical devices, biological profiles, and personal effects, that promote a multidisciplinary view that render reports on personal identification as a more integrated process than just a single technique. Additionally, virtual 3 D models obtained from imaging devices (CT scanners and intraoral surface scanners), photogrammetry [36], and/or open software [37] are also proving to be valuable tools for reconciliation of antemortem and postmortem data. Their use is quite promising, considering the lack of specialised imaging equipment in most of the DML units. The cooperation between SAF and the Scientific Police of Paraíba state (Northeast Brazil) even helped establish a positive identification, leading to the arrest of suspects [38].

Nonetheless, “routine” casework solely focused on postmortem examinations is, at best, omissive of the real scope of forensic work on personal identification. Thus, PCI recently took initiatives to address the issue of missing persons.

### Missing persons in Santa Catarina

Many indicators suggest that the disappearance of persons is a serious and widespread problem in Brazil, and many cases appear to intersect with urban violence [39]. Nearly 63 000 persons were reported missing in 2020, and even though about 31 000 were reportedly located [13], initiatives to address the issue are still localised and not very interconnected [39].

Recent national legislation recognised a broader concept of the missing persons situation, making explicit the state’s obligation to search and locate them [40], complementing the previous norm [41–44]. The newly instituted National Policy for Missing Persons [45], coordinated by the Ministry of Justice and Public Safety, formed working groups that include forensic anthropology and forensic odontology to improve its promotion [16, 46]. In a historical action, a national campaign was promoted in June 2021 to collect DNA from relatives of missing persons, which managed to gather samples from 1 620 families. This material is currently being processed for addition into the national DNA databank, and its comparisons with previously registered profiles from unknown human remains have already helped identify at least 18 of these persons [45, 47].

In 2020, Santa Catarina had large numbers of persons not only reported missing (3 285), but also located (3 722), and a missing persons rate per 100 000 inhabitants (45.3) above national average (29.7) [13]. Specific legislation on missing persons was already passed by the state in 2015 [48], determining the creation of a database managed by the Civil Police’s Missing Person Police Agency (DPPD), the obligation of DML to inform the reception of unknown human remains, the collection of DNA to form the state’s DNA databank, and, if feasible, the collection of dental data. These are in addition to determining the immediate and everlasting investigation of cases reported to the police.

DPPD is responsible for investigating cases, referring families for reference DNA sampling at PCI, and managing the official database of missing persons, which contains their personal data, descriptions, and images. Some of these data are available to the public on a website, allowing the user to filter local disappeared and reappeared persons by age, sex, date, and location last seen [49]. Another police stakeholder is the Military Police’s *SOS Desaparecidos* service, which functions as an additional channel for reporting missing persons and broadcasting their information, including on local TV news. It also contains a database of missing and located persons online [50], where one can consult similar data as above. The Public Ministry, through its Location and Identification Programme for the Disappeared (PLID), the local representant of the National System for the Localization and Identification of the Disappeared (SINALID), is responsible for compiling data on missing persons in Santa Catarina from different agencies [51]. The SINALID database, available only upon request and approval for public servants only, holds national data on missing persons, institutionalised persons, and unidentified human remains. PCI submits all data about unknown human remains from suspicious or violent deaths to DPPD, but does not make any information about the cadavers examined available to the public. Finally, families are represented by the independent, non-profit civilian association Support Group for Families of the Disappeared (GAFAD) [52], which orients and refers relatives to social, psychological, and legal assistance necessary to withstand the process of searching for their loved ones. GAFAD’s website is a database that is managed by volunteers, and displays several posters of missing persons from information provided by relatives.

Although aimed at addressing the same issue, stakeholders in Santa Catarina produce and manage data to some level, but still do not fully nor equally share the information. Postmortem data are legally transmitted by PCI to DPPD, but the official missing persons database is not readily accessible to other public safety institutions. This precludes PCI from antemortem versus postmortem conciliation procedures aimed at formulating identity hypothesis. The lack of integration at local and national levels is reported [14, 39], and such context in Santa Catarina motivated PCI to take an initiative to try to promote improved levels of integration between stakeholders. About 7 months prior to the national campaign for DNA collection, PCI’s DALF, DML, and Direction of Institute of Civil and Criminal Identification (DICC) cooperated with all the institutions mentioned to conduct a similar initiative through “*Programme* *Conecta*—*perícia conectando famílias*”.

Conecta is an integrated and multidisciplinary programme of PCI with the objective of promoting the location and identification of missing persons by using personal data and DNA profiles voluntarily provided by their families. Joint efforts convene and schedule families for reference DNA sample collection, similarly to the national campaign. The highlight of Santa Catarina’s Programme is that in the same appointment, relatives are also interviewed by PCI staff trained by SAF on antemortem data collection. Additionally, cases of disappearances that took place a long time ago are also referred to DICC staff that can produce age progression composites, providing an “updated” facial image of the missing person that can be published by relatives [53].

Conecta’s first actions took place in Florianópolis in November 2020 and February 2021 [54]. For the national campaign’s efforts, Conecta’s *modus operandi* was kept in the capital, and a new capacitation course for PCI staff allowed it to be expanded to the other five major PCI units across the state. In total, 33 families were interviewed and 63 DNA samples were collected. The programme established a positive precedent. Although 33 families may not seem like many, convening “genetically eligible” families is not a simple task. Legally accepted and statistically significant pedigree trees demand at least two relatives in the following order: both parents; one parent, a spouse, and their offspring; spouse and offspring; one parent and a sibling; two or more biological siblings; or an identical twin [48]. On 18 August 2021, the first missing person was identified in Santa Catarina using a sample collected by the Conecta Programme during the national campaign (Clineu Uehara, personal communication).

The programme has improved and expanded its reach, as the service stations across the state are still available for families to reach out. Despite being one of the smallest states in Brazil, Santa Catarina has the fifth largest missing person’s reference DNA database in Brazil [55].

## DVI

In the past years, Santa Catarina has had many natural and some non-natural disasters that have taken multiple lives. The largest death toll of a non-natural tragedy in the state reached 43 after a multiple automobile accident in Joinville city [56]. In 2008, heavy rains and over 4 000 landslides took 143 lives in the Itajaí Valley area [57]. Historically, the management of these dead bodies, from the scene to the morgue and then back to their families, has been done effectively by PCI, but without adherence to international DVI standards. Following December 2020s storms and floods in the Presidente Getúlio city area [58], all 21 fatal victims were successfully identified, but PCI administration felt that DVI operations still needed to be improved. Thus, they created a temporary working group to structure DVI actions at an institutional level [59].

The working group is presided by a SAF member and formed by other 20 PCI staff from different sectors. It has been adapting INTERPOL’s DVI Guide and other DVI manuals from other Brazilian Scientific Police agencies to the local context. The suggestion for the creation of a permanent DVI commission and the constitution of a DVI unit through specialised training are among the initiatives taken by the working group. Recently, the Brazilian Army’s 5th Humanitarian Force Exercise laid the ground for PCI’s first DVI training on Phase 1 procedures. After a preparatory course, a landslide with multiple deaths set in Blumenau city was simulated. Then, 30 students, encompassing PCI staff and the military, processed the scene following INTERPOL’s DVI procedures. DVI doctrine was also promoted along other institutions attending the exercise (Civilian Defence, Firefighter Corps, Military and Civil Police), allowing for a better understanding of the needs and objectives of forensic work in future disasters [60].

### Current challenges and future directions

Despite being a positive first step towards improving forensic anthropology and forensic odontology in Santa Catarina, the operations of SAF still face major challenges. It remains quite short-staffed, with only one forensic odontologist, one forensic pathologist, and one medicolegal assistant, all of whom still need to work part-time on parallel activities within the institution. A technical cooperation with the Federal University of Santa Catarina’s (UFSC) Departments of Morphology, Odontology and Archaeology is expected to be approved briefly, enabling not only an increase of human resources, but also a prospective, research-oriented view of SAF’s casework aimed at providing better indicators for public safety policies.

In the meantime, because of short staffing, the removal and transport of skeletal and biological material from most scenes is still not supervised by specialised personnel. Notably, crime scene analysis is performed by forensic analysts, and the recovery of virtually all skeletal remains is done without archaeological technique. As DML’s official experts usually do not go to crime scenes, proper routine search and recovery of skeletal or fragmented remains currently demand a great deal of cooperation between IC staff, Civil and Military Police. However, it is often impossible to achieve this because of numerous factors, ranging from no specific training of crime scene professionals on anthropological/archaeological body recovery procedures to the lack of human and material resources from PCI and the police agencies to preserve and secure scenes. Nonetheless, whenever possible, SAF staff will accompany teams on the ground to try to promote improved search and recovery of human remains.

Understaffing also clearly hampers the time needed to prepare skeletal material, conduct analysis, and file the reports. Moreover, it also hinders the sector from enacting management mechanisms to control cognitive bias, because contextual information about the cases is inevitably disclosed to the staff when the need for a specialised examination is verified. Additionally, short staffing can hinder the blind peer-reviewing of reports. A significant backlog of previous cases of skeletal material not examined by SAF is still housed in DML units across the state, which also presents a secondary challenge.

Despite these difficulties, recent improvements for routine casework are quite significant. They not only optimise resources available for genetic examinations, but ultimately benefit mourning families by providing them with some relief when identifications are more rapidly concluded.

Although there have been important advances at the local and national levels, the missing persons policy in Santa Catarina still has significant room for improvement. Stakeholders have gradually begun to improve collaboration, but the persistent lack of integration of databases is an apparent major challenge to effectively implement policy. Furthermore, PCI’s lack of automated database software for conciliating available dental and anthropological information on human remains and missing persons also represents a major constraint that jeopardises the management of all data collected. Nevertheless, Conecta Programme’s integrated and multidisciplinary initiative, as well as its later expansion in congruence with the national efforts, serves as a promising model for good practices in Brazil.

Even with a history of severe disasters state-wide, the adaptation of DVI good practices to the local context is yet to be improved. Guidelines are still being developed and a specialised unit is still in formation. Expansion of a continuous and specific training programme for staff of all units could be a starting point to consolidate this long-needed improvement. Even so, following the latest simulation, PCI’s upgraded integration with the other responding institutions signals better preparation for disasters ahead.
